# Generation and functional characterization of tuft cells in non-human primate pancreatic ducts through organoid culture systems

**DOI:** 10.3389/fcell.2025.1593226

**Published:** 2025-05-06

**Authors:** Kosuke Sakaguchi, Chiemi Kimura-Nakajima, Akihiko Inaba, Yoshiko Hatano, Hanako Ogawa, Yuichi Koshiishi, Keisuke Tanaka, Tatsuya Kometani, Makoto Ohmoto, Koji Sato, Hiroo Imai, Ken Iwatsuki

**Affiliations:** ^1^Faculty of Applied Bioscience, Tokyo University of Agriculture, Tokyo, Japan; ^2^Graduate School of Science, Osaka University, Suita, Ōsaka, Japan; ^3^Faculty of Health and Welfare, Takasaki University of Health and Welfare, Takasaki, Gunma, Japan; ^4^Department of Applied Biological Chemistry, The University of Tokyo, Tokyo, Japan; ^5^Center for the Evolutionary Origins of Human Behavior, Kyoto University, Kyoto, Japan

**Keywords:** tuft cells, primate, organoid, pancreas, type 2 immunity

## Abstract

The pancreatic duct plays a key role in collecting pancreatic juice, which is rich in digestive enzymes. The fluid flows unidirectionally into the duodenum, where it mixes with partially digested food to further facilitate digestion. In this study, we report the generation of pancreatic ductal organoids from non-human primates for the first time, aimed at investigating the role of tuft cells that reside in the pancreatic duct since no studies have addressed the role of tuft cells in the pancreas. The organoids were maintained in a medium supplemented with Wnt3a, Noggin, R-spondin, and other factors that support pancreatic duct proliferation. These pancreatic organoids expressed the stem cell marker LGR5 mRNA and the ductal marker protein CK19, although tuft cell markers were not detectable at this stage. Upon stimulation with IL-4/13, tuft cell differentiation was confirmed by immunohistochemistry and transcriptomic analysis. We observed induction of DCLK1, as well as taste signaling molecules such as TRPM5 and PLCβ2, which are markers of type II taste cells. Additionally, upregulation of LYZ and DEFB1 mRNA indicated the expression of antimicrobial peptide markers, alongside molecules associated with inflammation. Furthermore, the differentiated organoids specifically responded to a bitter compound, suggesting that pancreatic tuft cells may play a role in detecting potentially harmful chemicals. Finally, immunohistochemical analysis identified tuft cells in the non-human primate pancreas, supporting their involvement in sensing harmful compounds and regulating protective responses within the pancreas.

## Introduction

The pancreas serves as both an endocrine and exocrine organ, playing a crucial role in maintaining the body’s homeostasis (Hu et al., 2025). The endocrine function is carried out by the endocrine cells (α, β, and δ cells) of the islets of Langerhans, which regulate blood glucose levels. The exocrine function is performed by acinar cells, which secrete digestive enzymes that are collected by the pancreatic ducts and mixed with the alimentary bolus. Although pancreatic duct cells are a potential origin of pancreatic cancer cells (Storz and Crawford, 2020), their role beyond being a conduit for pancreatic fluids, such as their potential function as sensors for various signals or stimuli, remains poorly understood.

Recently, pancreatic ductal organoids have been successfully established in both mice and humans (Huch et al., 2013; Broutier et al., 2016). These ductal organoids possess the ability to differentiate into both endocrine and ductal cells (Huch et al., 2013; Loomans et al., 2018). In a previous study, we showed both *in vitro* and *in vivo* that pancreatic duct-derived organoids differentiate into Ngn3-positive cells, which are precursors of endocrine cells, using an Ngn3-EGFP transgenic mouse model (Nakajima et al., 2018; Kimura-Nakajima et al., 2021). Additionally, we observed the mRNA expression of intestinal peptide hormones and transcription factors that function downstream of Ngn3. Notably, we also detected the mRNA expression of tuft cell lineage markers, such as Pou2f3, Gnat3 (alpha-gustducin), and Trpm5, during the differentiation process of ductal organoids (Kimura-Nakajima et al., 2021). Since there are no reports to date demonstrating the transition of pancreatic ductal stem cells into tuft cells, this observation prompted us to consider whether tuft cells could be generated from the pancreatic duct, as has been observed in endocrine lineages.

Tuft cells, which are sometimes referred to as brush cells or solitary chemosensory cells (Sbarbati and Osculati, 2005; Schneider et al., 2019), are found in the epithelial cells of various tissues and play roles in bioprotective activities. Tuft cells were initially characterized by their unique morphology and the expression of taste-related molecules, such as Gnat3 (alpha-gustducin), Plcβ2, Trpm5, and bitter taste receptors (Hofer et al., 1996; Hofer and Drenckhahn, 1998; Kaske et al., 2007; Lin et al., 2008). Through the taste signaling pathway, tuft cells likely transduce chemical signals to nearby neurons or immune cells, mediating the elimination of unwanted microorganisms or noxious stimuli. Indeed, tuft cells are known to produce IL-25, leukotrienes, prostaglandins, and choline acetyltransferase (ChAT), a key enzyme in acetylcholine production, underscoring their role in immune responses and the exclusion of harmful substances (Tizzano et al., 2010; Krasteva et al., 2011; Saunders et al., 2014; Gerbe et al., 2016; Howitt et al., 2016; Nadjsombati et al., 2018; O'Leary et al., 2019; Schutz et al., 2019; Billipp et al., 2021). However, since there has been no reliable *in vitro* culture system to analyze the immune system, the precise function of tuft cells in the pancreas remains unknown.

Rodents are commonly used as experimental animals, including genetically engineered models, and are valuable for studying cellular functions. In tuft cell research, mouse models have been employed to investigate their functional role in response to microorganisms and hazardous compounds (Tizzano et al., 2011; Gerbe et al., 2016; Howitt et al., 2016). However, since tuft cells are infrequent, in addition to strain difference in the number of tuft cells in the pancreas, characterizing them in the mouse pancreas is challenging (DelGiorno et al., 2020b). Additionally, the selectivity of taste receptors that may “sense” chemical substances differs between rodents and humans, making it difficult to speculate on what tuft cells might detect in humans (Nelson et al., 2002; Toda et al., 2013; Hayakawa et al., 2014; Lossow et al., 2016). Therefore, it may be beneficial to introduce other animal models that more closely mimic human physiology. Given the considerable logistical and ethical constraints in obtaining human tissues, non-human primates, which possess taste receptor functions similar to those of humans, were selected as a suitable animal model.

In this study, we demonstrate the generation of pancreatic duct organoids from non-human primates, which can be induced to differentiate into tuft cells by IL-4/13. These cytokines act through a shared receptor subunit, IL-4Rα, and are central to the type 2 immune response, which plays a key role in host defense against parasitic infections and contribute to allergic responses (Bernstein et al., 2023). We observed the expression of taste-related molecules, including taste receptors, essential for taste signal transduction, as well as molecules involved in biological defense in differentiated tuft cells. We also investigated whether differentiated tuft cells have the potential to sense noxious compounds that may arise from duodenal reflux or pancreatic ductal reflux. Finally, we identified that tuft cells reside within the pancreatic tissue of macaques, extending from Vater’s papilla to the tail of the duct.

## Materials and methods

### Animals

Macaques (*Macaca mulatta* and *Macaca fuscata*, 1–22 years old) was used for the experiments. The study was approved by Kyoto University (Approval No. 2019-039, 2020-002, 2021-007, 2022-036, 2023-136), based on the Guidelines for Care and Use of Nonhuman Primates of the Primates Institute, Kyoto University (Version 3, 9 June 2010).

Ngn3-GFP mice (Kimura-Nakajima et al., 2021), Pou2f3-knockout (KO) mice (Matsumoto et al., 2011), and Wild-type mouse (7–10 weeks old) used in this study were housed, cared for, and used in accordance with the Guiding Principles in the Care and Use of Animals published by the Animal Care Committee of Tokyo University of Agriculture (Approval No. 2020080) and the Animal Experiment Committees of the Takasaki University of Health and Welfare, Faculty of Health and Welfare (Approval No. 2101).

### Administration of mice with succinate

Wild-type and Pou2f3-KO mice were treated with or without 100 mM succinate (FUJIFILM Wako, Japan) in water for 7 days. After perfusion fixation with 4% paraformaldehyde, the pancreas and ileum were harvested. Additionally, these tissues were post-fixed in 4% paraformaldehyde (Nacalai Tesque, Japan) for 2 h on ice.

### Organoid culture media

The proliferation medium (PRO) is a modified version of the medium described by Broutier et al. (2016). It consists of a basal medium supplemented with 1:50 B27 supplement (without vitamin A; Thermo Fisher Scientific, United States), 1:100 N2 supplement (Thermo Fisher Scientific), 1 mM N-acetylcysteine (Sigma-Aldrich, United States), 30% Wnt3a-conditioned medium (lab-made), 5% R-spondin2-conditioned medium (lab-made), 10 mM nicotinamide (FUJIFILM Wako, Japan), 10 nM recombinant human [Leu15]-gastrin1 (Sigma-Aldrich), 50 ng/mL recombinant human EGF (Sigma-Aldrich), 100 ng/mL recombinant human FGF10 (Peprotech, United States), 25 ng/mL recombinant human Noggin (Proteintech), 500 nM A83-01 (FUJIFILM Wako), 100 ng/mL recombinant human IGF-1 (FUJIFILM Wako), and 50 ng/mL recombinant human FGF-2 (FUJIFILM Wako). The basal medium consists of Advanced DMEM/F-12 (Thermo Fisher Scientific) supplemented with 1× Gentamicin/Amphotericin B (Thermo Fisher Scientific), 2 mM L-Alanyl-L-Glutamine (Nacalai Tesque), and 10 mM HEPES (Nacalai Tesque). Wnt3a-conditioned medium was prepared using L1 cells, and R-spondin2-conditioned medium was prepared using HEK293T cells. For differentiation into tuft cells, nicotinamide and EGF were removed, and 40 ng/mL recombinant human IL-4 or IL-13 (Peprotech) was added.

### Establishment of macaque pancreatic ductal organoids

Macaque pancreatic ductal organoids were established as previously described (Broutier et al., 2016), with slight modifications. Briefly, the macaque pancreas was dissected, minced into small fragments, and repeatedly washed with wash medium. The samples were then incubated on a shaker at 37°C for 1 h in a digestion solution containing wash medium supplemented with 0.25 mg/mL collagenase IV (Roche Diagnostics, Switzerland), 0.25 mg/mL dispase II (Roche Diagnostics), and 0.1 mg/mL DNase I (Sigma-Aldrich). The isolated ducts and pancreatic tissues were embedded in Matrigel (CORNING, United States) and overlaid with proliferation medium. The medium was refreshed every 2 days. Organoids were passaged every 10–14 days using TrypLE Express (Thermo Fisher Scientific, United States). For the first 4 days of culture, 10 mM Y-27632 (Nacalai Tesque) was added to the medium. Mouse pancreatic ductal organoids were prepared as previously described (Kimura-Nakajima et al., 2021).

### Quantification analysis of tuft cells in pancreas tissues and organoids

The macaque pancreas tissue was sectioned into the papilla of Vater, and the head, body, and tail regions of the pancreas. Frozen sections were prepared from each region, and DCLK1-positive cells were quantified in three sections from each region. In murine tissues, Dclk1-positive cells were quantified from the papilla of Vater to the common pancreatic/bile duct (Dolensek et al., 2015), whereas in the ileum, Dclk1-positive cells were quantified in 10 villi of wild-type mice (*n* = 3), Pou2f3-KO mice (*n* = 2). DCLK1-positive cells were quantified in macaque pancreatic ductal organoids (≥150 μm in size) under three experimental conditions: PRO (*n* = 6), IL-4 (*n* = 12), and IL-13 (*n* = 15).

### Immunostaining of tissues and organoids

Pancreatic tissues were fixed in 4% paraformaldehyde for 90 min at 4°C, washed with PBS, cryoprotected in 30% sucrose, embedded in OCT compound (Sakura Finetek, Japan), and frozen. Cryosections (12–16 µm) were fixed, washed, and blocked with 0.3% Triton X-100% and 2% donkey serum for 1 h. Sections were incubated overnight at 4°C with primary antibodies, followed by 1-h incubation with secondary antibodies. Nuclear staining was done using DAPI (Nacalai Tesque).

For organoid immunostaining, organoids were fixed in 4% paraformaldehyde for 30 min, washed, blocked overnight at 4°C, and incubated with primary and secondary antibodies. Nuclear staining was performed with Hoechst 33342. Fluorescence images were acquired using a confocal microscope (FV-1200; Olympus, Japan).

Primary antibodies: rabbit anti-CK19 antibody was provided by Dr. Miyajima A. (The University of Tokyo), rabbit anti-pancreatic alpha amylase (ab21156, Abcam), rabbit anti-DCAMKL1 (ab31704, Abcam), goat anti-GNAT3 (ab113664, Abcam), goat anti-Choline Acetyltransferase (AB144, Chemicon), rabbit anti-TRPM5 (18027-1-AP, Proteintech), mouse anti-Cytokeratin 19 (ab9221, Abcam), rabbit anti-Chromogranin A (ab15160, Abcam), and rat anti-CD326 (Ep-CAM) (552370, BD Pharmingen). Secondary antibodies: Alexa Fluor 488 donkey anti-rabbit IgG (A21206, Invitrogen), Alexa Fluor 555 donkey anti-goat IgG (A21432, Invitrogen), Alexa Fluor 555 donkey anti-mouse IgG (A31570, Invitrogen), and Alexa Fluor 594 donkey anti-rat IgG (A21209, Invitrogen).

### RNA-seq and statical analysis

Total RNA was extracted using ISOGEN (Nippon Gene) following the manufacturer’s instructions and assessed for quality with a Bioanalyzer (Agilent Technologies). cDNA libraries were prepared from 50 ng of total RNA using the NEB Next Ultra II RNA Library Prep Kit (New England Biolabs). The accurate concentration of each library was measured with the KAPA Library Quantification Kit (Kapa Biosystems). Sequencing was conducted on the NextSeq 500 (Illumina) with 75-bp single read, and sequence data were generated in Fastq format using bcl2fastq version 2.18.0.12. The read data are available in the NCBI BioProject (ID: PRJNA1231724 for the monkey and ID: PRJNA1234775 for the mouse).

Reference genome information for rhesus macaque (*M. mulatta*; GCF_003339765.1_Mmul_10) was obtained from NCBI Genome (https://www.ncbi.nlm.nih.gov/genome). Adapter sequences and low-quality reads were removed using CLC Genomics Workbench 20 (Qiagen), and clean reads were mapped by the default parameters to the reference genome. Gene expression clustering was visualized using principal component analysis (PCA) and heatmap with the R packages “prcomp” and “pheatmap” (Figure 3F, FC ≥ 2 and FDR p-value <0.05).

MA-plot was generated by averaging count data after TPM normalization, calculating fold change relative to the PRO group, and plotting log2-transformed values with the R package “ggplot2”. Statistical significance was assessed using a one-way ANOVA (p < 0.05), followed by Tukey’s honest significant difference (HSD) test for TPM comparisons.

### Calcium imaging

Organoids cultured with the media with IL-4 were used for the assay. Organoids were dissociated with TrypLE Express (Thermo Fisher Scientific), seeded onto poly-L-lysine- and laminin-coated glass, and incubated in PRO till cell attached to the glass. Prior to calcium imaging, cells were loaded with 2.5 µM Fura2-AM (Dojindo, Japan) in PRO for 30–50 min, washed and perfused with Ringer’s solution (30 mM NaCl, 115 mM *N*-methyl-*D*-glucamine, 5 mM KCl, 2 mM CaCl_2_, 1 mM MgCl_2_, 10 mM glucose, 10 mM HEPES, and 1 mM sodium pyruvate, pH 7.4) at a flow rate of 30 μL/s and stimulated with 10 mM denatonium (FUJIFILM Wako) and 20 µM ATP solution for 10 s. U73122 (Cayman Chemical, USA) was incubated at 40 µM for 10 min. Fluorescence images were acquired using Leica’s LAS X software with excitation wavelengths of 340 nm and 380 nm, emission detected at 510 nm. Leica’s LAS X software was used to determine a fluorescence ratio at 340 and 380 nm. Fluorescence was recorded using a CCD camera (DFC365 FX; Leica Microsystems, Germany) mounted on a Leica DMI6000B inverted microscope (Leica Microsystems).

## Result

**FIGURE 1:**
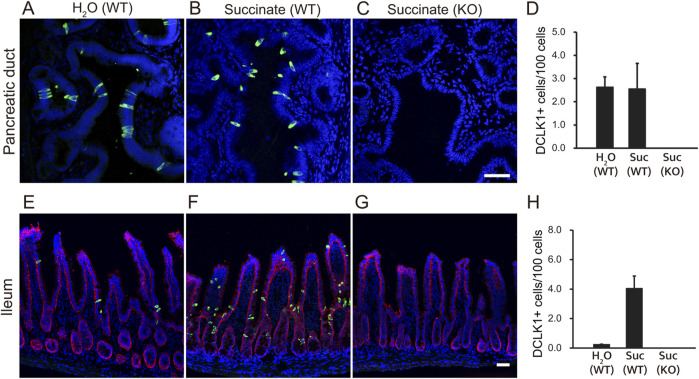
Tuft cells in the pancreatic ducts and small intestine of mice. Wild-type (WT) mice were treated with 100 mM succinate or water (H_2_O) for 7 days. Immunofluorescence staining was performed to detect Dclk1 (green), a tuft cell marker, in the pancreatic duct **(A, B)** and ileum **(E, F)** of wild-type mice. Dclk1 expression was examined in both the pancreatic duct and ileum of Pou2f3-KO mice treated with 100 mM succinate for 7 days **(C, G)**. Small intestinal epithelial cells were stained with Ep-CAM (red). Nuclei were stained with DAPI (blue). Scale bars: 50 µm. The number of Dclk1-expressing tuft cells in each condition was quantified **(D**, **H)**. Data are presented as means ± SEM (*n* = 3).

It has been almost 3 decades since tuft cells were first identified in the rat pancreatic duct (Hofer and Drenckhahn, 1998). Subsequent studies have shown that tuft cells are rare in the mouse duct (DelGiorno et al., 2020b). We re-examined this using immunohistochemistry with anti-Dclk1 (Doublecortin-like kinase 1) antibodies and confirmed that tuft cells are indeed rare in both the mouse pancreas and intestines (Figures 1A,E). To assess whether Dclk1-labeled tuft cells would increase their numbers upon microbial metabolites as shown previously (Nadjsombati et al., 2018), we administered succinate to mice, as it strongly promotes the expansion of tuft cells. Our results showed that succinate stimulation led to an increase in Dclk1-labeled populations in the ileum (Figures 1E,F), whereas no change was observed in the number of Dclk1-labeled cells in the pancreatic duct (Figures 1A,B). As a negative control, the absence of tuft cells was confirmed in Pou2f3-KO mice (Figures 1C,G). The number of Dclk1-labeled cells was quantified, expressed as a percentage, and used to generate the graph (Figures 1D,H).

Our previous transcriptome study indicated that mouse pancreatic duct stem/progenitor cells differentiate into both endocrine and tuft cells (Kimura-Nakajima et al., 2021). To replicate our previous experiments, we investigated whether mouse pancreatic duct organoids could differentiate into tuft cells upon withdrawal of nicotinamide from the culture medium. We observed an increase in tuft cell number, which was confirmed by the upregulation of Dclk1 and Pou2f3, key markers associated with tuft cell development (Supplementary Figure S1).

**FIGURE 2:**
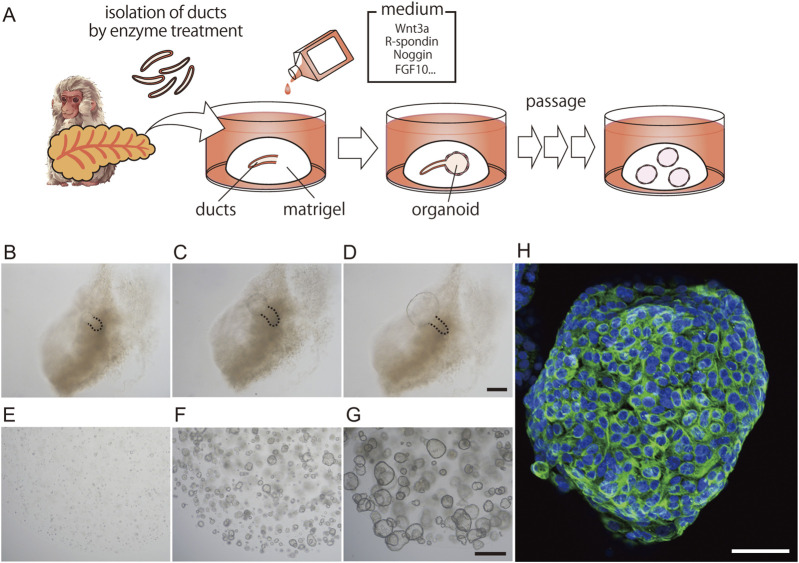
Generation of macaque pancreatic ductal organoids. Schematic illustration depicting the process of generating pancreatic ductal organoids **(A)**. Representative images of primary organoid cultures at days 2, 4, and 5 **(B–D)** and clusters of growing organoids at days 2, 5, and 8 after passage **(E–G)**. Day 10 pancreatic ductal organoids cultured with proliferation medium were stained with anti-CK19 antibodies (green, **(H)**. Nuclei were stained with Hoechst 33342 (blue). The pancreatic ducts are outlined with dashed lines. Scale bars: B-D, 200 μm; E-G, 500 μm; H, 50 µm.

We then investigated whether this phenomenon also occurs in the primate organoid culture system. To this end, we generated primate pancreatic duct organoids from macaques. Pancreatic ducts were isolated from the pancreas, embedded in Matrigel, and overlaid with a medium containing Wnt3a, R-spondin2, Noggin, EGF, and other supplements, as described in the Materials and Methods (Figure 2A). In the proliferation (PRO) medium, spherical structures, referred to as ductal organoids, were first observed at the edge of the ducts 2–4 days after culture (Figures 2B,C). Organoids were clearly distinguishable by Day 5 (Figure 2D). These ductal organoids could be passaged every 10–14 days (Figures 2E–G), cultured repeatedly for over 6 months, and stored frozen for future use.

To determine whether the proliferating cells were duct-derived, we performed whole-mount immunohistochemistry using antibodies against the ductal marker CK19. All cells within the organoids were CK19-positive (Figure 2H). However, we were unable to detect any tuft cell or endocrine cell markers, including DCLK1, Chromogranin A (CHGA), or Amylase (AMY), in the organoids cultured in PRO medium, despite using antibodies specific to each marker (Supplementary Figures S2A–C). In contrast, these differentiated cell markers were detected in macaque pancreatic tissue sections using the same antibodies (Supplementary Figures S2D–F).

It has been reported that tuft cell numbers increase significantly upon exposure to IL-4 or IL-13 both *in vivo* and *in vitro* (von Moltke et al., 2016; Inaba et al., 2021), possibly due to tuft cell differentiation. Based on this, we aimed to induce the differentiation of mature tuft cells in macaque pancreatic duct organoids by supplementing the culture medium with IL-4 or IL-13 and withdrawing nicotinamide and epidermal growth factor (EGF) for 5 days. To determine whether macaque ductal organoids are capable of differentiating into tuft cells, we performed both immunohistochemical and transcriptomic analyses.

**FIGURE 3:**
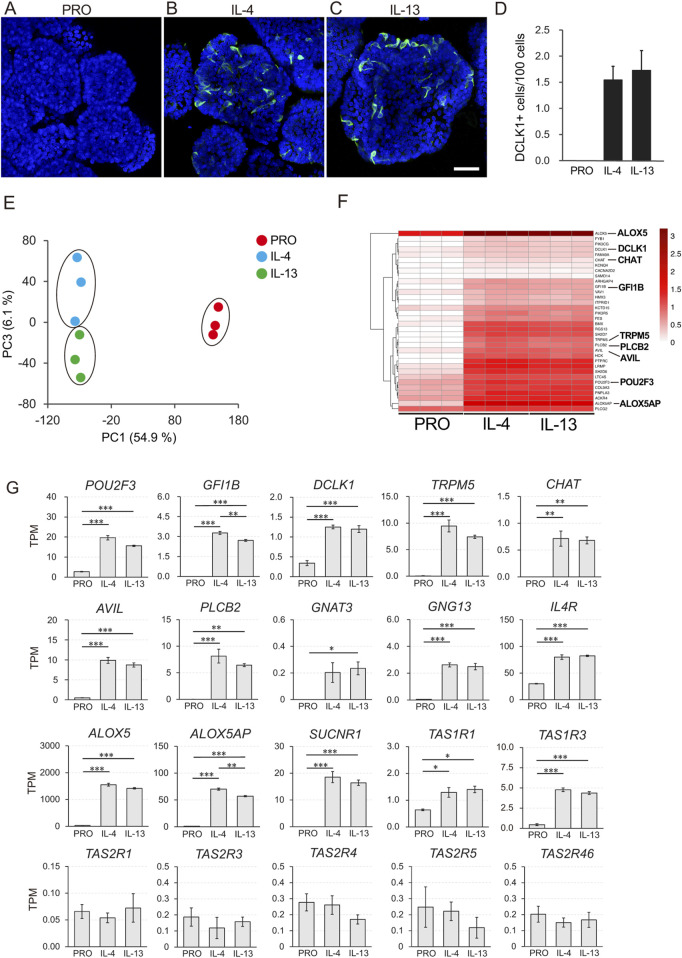
Transcriptome analysis of macaque ductal tuft cells induced by IL-4 and IL-13 stimulation. DCLK1 immunoreactive tuft cells were not detected before induction with IL-4 or IL-13 **(A)**. After stimulated with IL-4 or IL-13 for 5 days, DCLK1-expressing tuft cells (green) were observed **(B, C)**. Nuclei were stained with Hoechst 33342 (blue). Scale bars: A-C, 100 µm. Quantitative analysis of the DCLK1-expressing tuft cells under three culture conditions **(D)**. Principle component analysis (PCA) showed that individual culture condition fell into the same cluster **(E)**. The heatmap illustrates the tuft cell markers that were significantly upregulated in organoids induced by IL-4 and IL-13 (FC ≥ 2 and FDR p-value <0.05) **(F)**. Color bars represent Log10-transformed TPM values **(F)**. Gene expression of representative tuft cell markers (*POU2F3, GFI1B, DCLK1, TRPM5, CHAT, AVIL, PLCB2, GNAT3, ALOX5,* and *ALOX5AP*), genes related to tuft cell function (*GNG13, IL4R*, and *SUCNR1*), and taste receptors (*TAS1R1, TAS1R3, TAS2R1, TAS2R3, TAS2R4, TAS2R5*, and *TAS2R46*) is shown **(G)**. All data are presented as means ± SEM (*n* = 3). The p-value was determined by a one-way ANOVA followed by Tukey’s HSD test (*P < 0.05, **P < 0.01, ***P < 0.001).

We observed that IL-4 and IL-13 stimulation induced the differentiation of DCLK1-positive tuft cells, whereas no such differentiation occurred in organoids cultured in PRO medium (Figures 3A–C). Compared to the PRO medium, the number of DCLK1-positive tuft cells significantly increased upon supplementation with either IL-4 or IL-13 (Figure 3D).

The RNA-Seq analysis comparing the three distinct culture conditions (PRO, IL-4, and IL-13) revealed clear differentiation into distinct clusters of cell groups, as demonstrated by principal component analysis (PCA) (Figure 3E). MA plot analysis showed that 1981 and 2029 genes were significantly upregulated, while 1569 and 1483 genes were significantly downregulated in organoids cultured with IL-4 and IL-13, respectively, compared to the PRO medium (|FC| ≥ 2 and FDR p-value <0.05) (Supplementary Figure S3).

To further investigate the genes expressed by differentiated tuft cells, we visualized the expression of upregulated genes, known to be mouse tuft cell markers, across the three culture conditions (PRO, IL-4, and IL-13) using heatmap analysis (Figure 3F, FC ≥ 2 and FDR p-value <0.05). Among the 34 tuft-related markers, most were upregulated in both the IL-4 and IL-13 supplemented groups. These markers include POU2F3, GFI1B, DCLK1, TRPM5, CHAT, Advillin (AVIL), PLCB2, GNG13 and succinate receptor 1 (SUCNR1), all of which are representative tuft cell markers; ALOX5 and ALOX5AP, important in leukotriene synthesis; taste 1 receptors TAS1R1 and TAS1R3, which are umami taste receptors, lysozyme (LYZ), and defensin beta 1 (DEFB1), along with PTGS1 which act as self-defense molecules. Most of the molecules were identified as significantly upregulated genes (Figure 3G; Supplementary Figure S4).

In contrast, the expression of the proliferation marker gene MKI67 and the stem cell marker leucine-rich repeat-containing G protein-coupled receptor 5 (LGR5) was downregulated (Supplementary Figure S4). Notably, we did not observe significant changes in the expression of taste 2 receptors (TAS2Rs) and IL-25.

**FIGURE 4:**
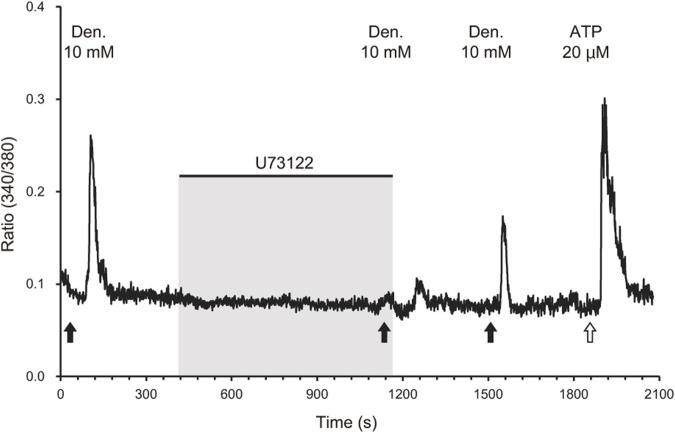
Phospholipase C-mediated calcium response of IL-4-treated organoids to a bitter compound. A representative waveform of a cell derived from IL-4-treated organoids exposed to 10 mM denatonium benzoate (black arrows) is shown. The response to denatonium benzoate was attenuated by the phospholipase C inhibitor U73122 (40 µM). The response recovered after the inhibitor was removed via perfusion for 6 min. Black bars indicate the timing of application of the phospholipase C inhibitor U73122 (40 µM) and following washing out. ATP (20 μM, a white arrow) was applied as a positive control to validate the cell viability.

Similar to tuft cells in the airways and intestines, we hypothesized that tuft cells in the pancreatic duct play essential roles in sensing chemicals that may be harmful to the body. To test this, we next examined whether the ductal organoids generated in this study are capable of detecting irritants or bitter compounds. We introduced a perfusion system for Ca^2+^ imaging using Fura2. We tested the bitter compound denatonium benzoate to observe the chemical responses of tuft cells in the organoid. We found that very few cells responded to 10 mM denatonium benzoate, and the response was inhibited by 40 µM of the phospholipase C (PLC) inhibitor U73122 (Figure 4). After washing out the perfusion buffer for 6 min, the response gradually recovered, suggesting that the denatonium response is specific to taste signaling. Additionally, we confirmed the viability of the cells by adding ATP to the same perfusion system. We did not observe any response to denatonium benzoate in organoids that were not subjected to tuft cell induction (Supplementary Figure S5).

**FIGURE 5:**
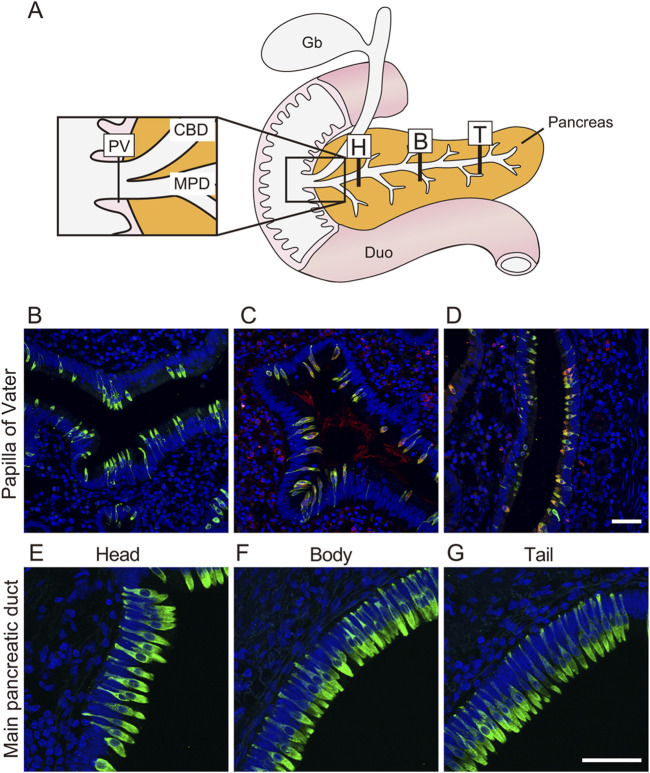
Tuft cells reside in the main pancreatic duct and papilla of Vater in monkey The schematic illustration shows location stained with anti-DCLK1 antibody **(A)**. Fluorescent immunostaining images of DCLK1-positive tuft cells through PV to Tail region **(B, E–G)**. Fluorescent immunohistochemical analysis show that DCLK1 (green) and CHAT (red, **(C)** or DCLK1 (green) and GNAT3 (red, **(D)** are partially co-localized around PV. Nuclei were stained with DAPI (blue). Scale bars: 50 µm. PV, papilla of Vater; CBD, common bile duct; MPD, main pancreatic duct; Gb, gallbladder; Duo, Duodenum; H, head of pancreas; B, body of pancreas; T, tail of pancreas.

Thus far, we have generated duct organoids from the pancreas, but the question of whether tuft cells reside within primate pancreatic ducts under normal physiological conditions remains unanswered. To address this, we sampled pancreatic ducts from macaques and investigated the presence of tuft cells along the pancreatic duct, from the papilla of Vater (PV) to the head and tail using immunohistochemistry (Figure 5A). Although some variation was observed among individual samples (Supplementary Table S1), we detected DCLK1-positive tuft cells along the pancreatic ducts (Figures 5B–G). Most tuft cells exhibited immunoreactivity to both DCLK1 and ChAT antibodies; however, not all GNAT3-positive cells co-localized with DCLK1, suggesting the existence of subtypes of tuft cells within the duct.

## Discussion

Over the past few decades, researchers have been seeking the origin of pancreatic stem cells that could give rise to both endocrine and exocrine lineages. In anticipation of the future therapeutic applications of these stem cells, increasing attention is being paid to the conditions that promote the emergence of endocrine cell progenitors and to their potential cellular origins. However, a significant debate still exists regarding the precise source of pancreatic stem cells.

Recent studies have presented conflicting evidence regarding the cellular origin of pancreatic β cells. One group has provided data suggesting that stem cells residing within the pancreatic duct have the potential to differentiate into β cells (Gribben et al., 2023). In contrast, another group has proposed that β cells may instead arise through transdifferentiation from exocrine cell (Magenheim et al., 2023). Both groups employed lineage tracing using genetically modified mouse models in conjunction with gene expression analyses. However, discrepancies in the experimental models and differences in data interpretation have precluded a definitive conclusion. As a result, the origin of β cells remains a matter of ongoing debate.

During the analysis of endocrine cells derived from mouse pancreatic organoids, we observed the expression of tuft cell markers in the same group of organoids that had differentiated into endocrine lineages (Kimura-Nakajima et al., 2021). Tuft cells have been shown to play a role in bioprotective activities across various tissues, with tissue-specific functions (O'Leary et al., 2019; Schneider et al., 2019). Although several decades have passed since the discovery of tuft cells in the pancreatic duct of rodents, the exact function of these cells within the duct remains uncertain. Analyzing tuft cell function is challenging due to their low abundance and the lack of reliable *in vitro* culture systems for studying them. Moreover, the specificity and selectivity of ligands for taste receptors vary among species, which limits the applicability of data obtained from rodent models. In this study, we focused on tuft cells present in the pancreatic ductal epithelium and, for the first time, successfully generated pancreatic ductal organoids from non-human primates. Since the original culture medium primarily supports cell proliferation within the organoids, differentiated cells were not observed. However, by removing nicotinamide and EGF from the medium and supplementing with IL-4 and IL-13, we were able to induce tuft cell differentiation.

It is well established that tuft cells in the intestine respond to parasitic infections by secreting IL-25, which alerts nearby lymphoid cells, specifically ILC2s (Kabata et al., 2018). In turn, ILC2s secrete IL-13, promoting the expansion of tuft cells as well as goblet cells, which subsequently secrete mucins to exclude parasites (Gerbe et al., 2016; von Moltke et al., 2016). Together with other studies, tuft cells are thought to play a self-defense role against external stimuli, although functions beyond self-defense have also been explored. Recently, tuft cells in the human intestine have been reported to serve as damage-induced reserve stem cells (Huang et al., 2024), suggesting that tuft cells in primates have multiple functional roles.

To speculate on the functions of pancreatic tuft cells, we investigated the changes in transcripts levels upon tuft cell propagation. Stimulation of ductal organoids with IL-4 or IL-13 led to both upregulation and downregulation of genes, with a comparable number and types of genes affected (Supplementary Figure S3). Additionally, an increased number of tuft cells was detected within the organoids, accompanied by the upregulation of SOCS1, SOCS2, and CISH, which are downstream molecules of STAT6 (Supplementary Figure S4) (Yoshimura et al., 2018). This strongly suggests that stem cells or progenitors in ductal organoids are activated by Janus kinase and signal transducers and activators of transcription (JAK-STAT) signaling pathway via the IL-4 receptor. It is interesting to explore how these signaling pathways influence tuft cell differentiation and proliferation, as STAT6 plays an essential role in the development of Th2 immunity (Kaplan et al., 1996). Utilizing gain-of-function (GOF) approaches in human disease models would be valuable for identifying the series of molecules regulated by STAT6, as STAT6-GOF patients exhibit upregulation of STAT6-targeted molecules (sturvey@bcchr.ca and Consortium, 2024).

Other molecules, such as ALOX5, ALOX5AP, ALOX15, PTGS1, LTC4S, PLA2G1B, PLA2G2A, PLA2G4D and PLA2G4G (Supplementary Figure S4), which serve as indicators of tuft cells and intermediate enzymes involved in leukotriene and prostaglandin production, were also upregulated following IL-4 and IL-13 stimulation. Leukotrienes and prostaglandins are critical signaling molecules that mediate immune responses involved in inflammation, allergy, fever, and other physiological processes (DelGiorno et al., 2020a; McGinty et al., 2020; Oyesola and Tait Wojno, 2021; Lee et al., 2025). These responses are essential for maintaining homeostasis in the body, although the precise triggers for these immune reactions remain poorly understood. For example, the specific ligands present on parasites in the intestines remain unidentified. Similarly, the molecules that tuft cells in the pancreatic ducts respond to in order to activate the immune system have yet to be determined. Furthermore, IL-4 and IL-13 induced pancreatic tuft cells appear to express IL-25 at lower levels than expected (Supplementary Figure S4), suggesting that an alternative pathway may be involved in the signal transduction by tuft cells to provoke immune responses.

The main pancreatic duct (MPD) joins the bile duct just before the ampulla of Vater in both humans and monkeys, where both ducts empty into the duodenum (Dolensek et al., 2015; Tsuchitani et al., 2016). This anatomical configuration renders the MPD susceptible to the influx of foreign substances from both the duodenum and the bile duct. It is well established that obstruction of the pancreatic duct, such as by a gallstone or duct ligation, can lead to the reflux of bile and pancreatic enzymes, which in turn can induce pancreatitis (van Geenen et al., 2010; Muili et al., 2013; Nakajima et al., 2018). Notably, bile acids contain bitter compounds, and gallstone-induced obstruction may result in the reflux of bile into the pancreatic duct. This phenomenon prompted us to investigate whether bitter compounds have an effect on tuft cells. The data presented in this study suggest that bitter compounds may act as a potential trigger for a response in the pancreatic duct (Figure 4). Denatonium is recognized as a bitter compound in both humans and mice, binding to the bitter taste receptors TAS2Rs (Behrens and Meyerhof, 2006), which transduce the bitter signal to the central nervous system. Multiple studies have demonstrated that tuft cells express taste receptors (Shah et al., 2009; Luo et al., 2019; Hollenhorst et al., 2022; Keshavarz et al., 2024), a finding that is consistent with the results of our study. Although we did not observe an upregulation of TAS2R genes when the organoids were induced with IL-4 and IL-13, we did detect mRNA expression of denatonium-sensitive receptors, particularly TAS2R4 and TAS2R46 (Meyerhof et al., 2010), in the ductal organoids. We hypothesize that the expression levels of TAS2Rs in these organoids may be too low to detect significant changes in expression, or that alternative receptors could be involved in the response to denatonium. It may be necessary to identify an improved method for inducing tuft cell differentiation using ductal organoids. Further studies are required to investigate these possibilities.

**FIGURE 6:**
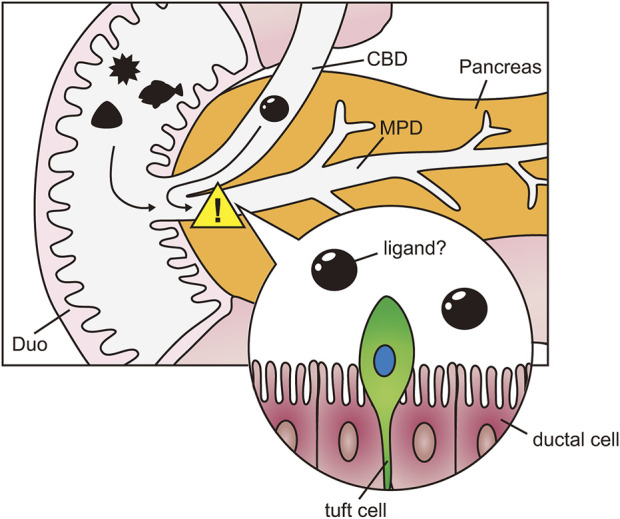
Speculation of tuft cell function in pancreas. Schematic diagram of the hypothesis that tuft cells in the pancreatic ducts detect harmful agents from the duodenum and bile ducts. Duo, Duodenum; CBD, common bile duct; MPD, main pancreatic duct.

The structure of the pancreas varies between humans and rodents (Dolensek et al., 2015), and non-human primates may serve as a more accurate model for human biology than mice. Compared to mice, macaque pancreatic ducts are notably enriched in tuft cells. Although our access to macaque sample is limited, our data suggest that tuft cell numbers are higher in macaques than in rodents, potentially reflecting species-specific functional differences. In this context, the use of non-human primates may provide valuable insights for future applications to human biology. Based on our findings, we speculate that the pancreatic ductal epithelium in primates is equipped with a self-defense mechanism mediated by tuft cells (Figure 6). While it is premature to draw definitive conclusions, tuft cells appear to perform their functions differently across various tissues, despite sharing similar sensory mechanisms.

Overall, we have successfully generated ductal organoids from macaque pancreatic ducts. In the present study, we tested only one differentiation condition, which selectively promoted tuft cell differentiation. However, as demonstrated in mouse models, endocrine lineages may also be induced with our culture model. Further studies are warranted to determine whether primate pancreatic ductal organoids possess bipotency to give rise to both tuft and enteroendocrine cells.

## Data Availability

The datasets presented in this study can be found in online repositories. The names of the repository/repositories and accession number(s) can be found in the article/Supplementary Material.
