# Supplementary material for: Generation and functional characterization of tuft cells in non-human primate pancreatic ducts through organoid culture systems
Source: Front Cell Dev Biol. 2025 May 6;13:1593226. doi: 10.3389/fcell.2025.1593226 (PMC12089129; doi:10.3389/fcell.2025.1593226)
Supplement: Supplementary file 1 [file DataSheet2.pdf]

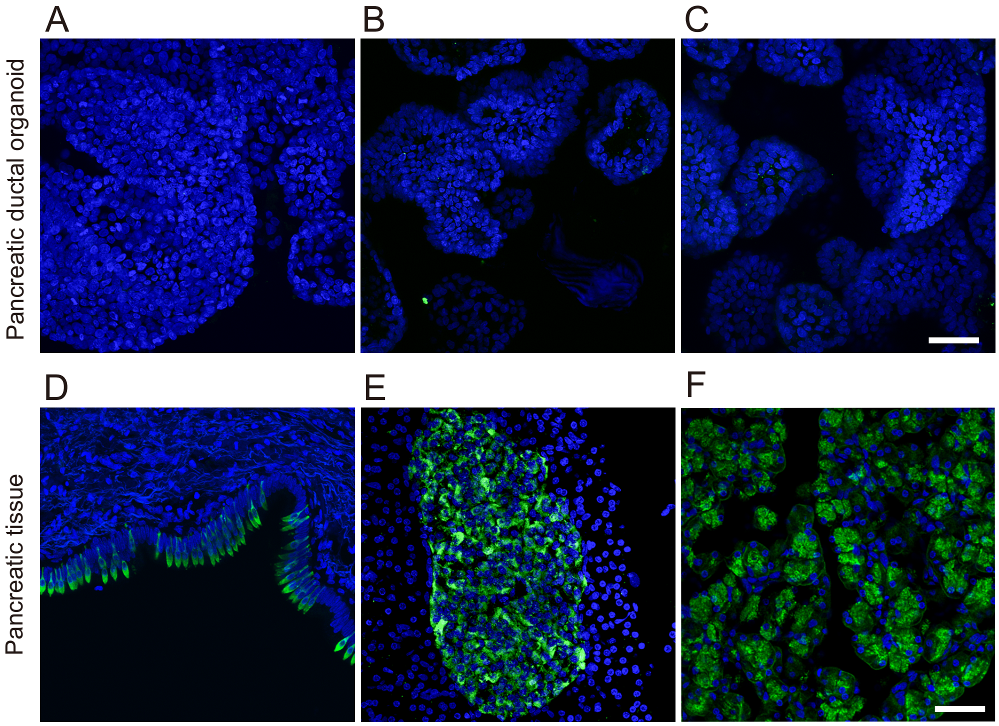

Supplementary Figure 2. Pancreatic ductal organoids cultured in PRO media exhibit immature phenotype. Immunofluorescence staining was performed to assess differentiated cell markers in pancreatic ductal organoids **(A–C)** and pancreatic tissues **(D–F)**. Antibodies targeting DCLK1, CHGA, and AMY were used to identify tuft cells (green: **A** and **D**), endocrine cells (green: B and E), and acinar cells (green: **C** and **F**), respectively. No expression of these mature cell markers was detected in ductal organoids cultured in PRO media. Nuclei were stained with Hoechst 33342 for organoids and DAPI for tissue sections. Scale bars: 50 µm.
