# Supplementary material for: Generation and functional characterization of tuft cells in non-human primate pancreatic ducts through organoid culture systems
Source: Front Cell Dev Biol. 2025 May 6;13:1593226. doi: 10.3389/fcell.2025.1593226 (PMC12089129; doi:10.3389/fcell.2025.1593226)
Supplement: Supplementary file 2 [file DataSheet4.pdf]

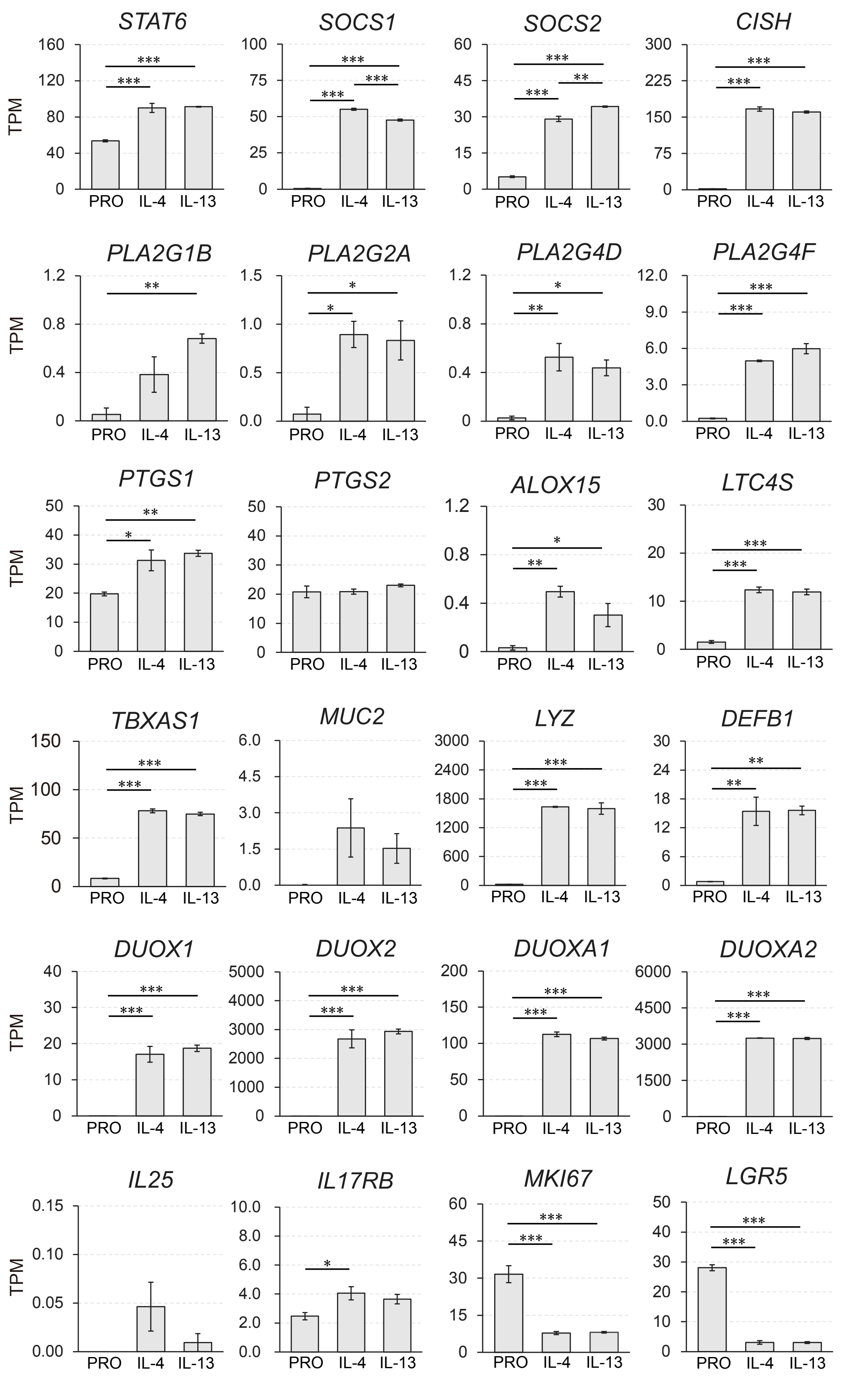

Supplementary Figure 4. Genes related to biological defense are upregulated in pancreatic ductal organoids treated with IL-4 and IL-13. Graphs show the expression levels of genes involved in immune response (*STAT6, SOCS1, SOCS2* and *CISH*), eicosanoid biosynthesis-related molecules (*PLA2G1B, PLA2G2A, PLA2G4D, PLA2G4F, PTGS1, PTGS2, ALOX15, LTC4S* and *TBXAS1*), mucosal immunity (*MUC2, DEFB1, LYZ, DUOX1, DUOX2, DUOXA1* and *DUOXA2*), tuft cell markers (*IL25* and *IL17RB*) and stem cell markers (*MKI67* and *LGR5*). Data are shown as means ± SEM ($n = 3$). * $P < 0.05$, ** $P < 0.01$, *** $P < 0.001$.
