# Supplementary material for: Generation and functional characterization of tuft cells in non-human primate pancreatic ducts through organoid culture systems
Source: Front Cell Dev Biol. 2025 May 6;13:1593226. doi: 10.3389/fcell.2025.1593226 (PMC12089129; doi:10.3389/fcell.2025.1593226)
Supplement: Supplementary file 3 [file Table1.docx]

**Supplementary Table 1.** Information on the monkeys used in the experiment and the proportion of tuft cells in their pancreatic ducts.

| **Species** | **Gender** | **Age** | **PV (%)** | **Head (%)** | **Body (%)** | **Tail (%)** |
| --- | --- | --- | --- | --- | --- | --- |
| *M.Mulatta* | Female | 2 years, 10 months | 1.38 | 6.23 | 11.5 | 8.76 |
| *M.Fuscata* | Male | 4 years, 11 months | 5.48 | 34.9 | 28.2 | 39.0 |
| *M.Fuscata* | Male | 7 years, 2 months | 8.84 | 3.2 | 0.06 | 0.07 |
| *M.Mulatta* | Female | 7 years, 3 months | 4.51 | 28.8 | 28.7 | 30.9 |
| *M.Mulatta* | Female | 20 years | 1.20 | 2.9 | 0 | 0.59 |
| *M.Mulatta* | Female | 22 years, 2 months | 8.07 | 13.7 | 17.8 | 11.5 |
