# Supplementary material for: Generation and functional characterization of tuft cells in non-human primate pancreatic ducts through organoid culture systems
Source: Front Cell Dev Biol. 2025 May 6;13:1593226. doi: 10.3389/fcell.2025.1593226 (PMC12089129; doi:10.3389/fcell.2025.1593226)
Supplement: Supplementary file 4 [file DataSheet3.pdf]

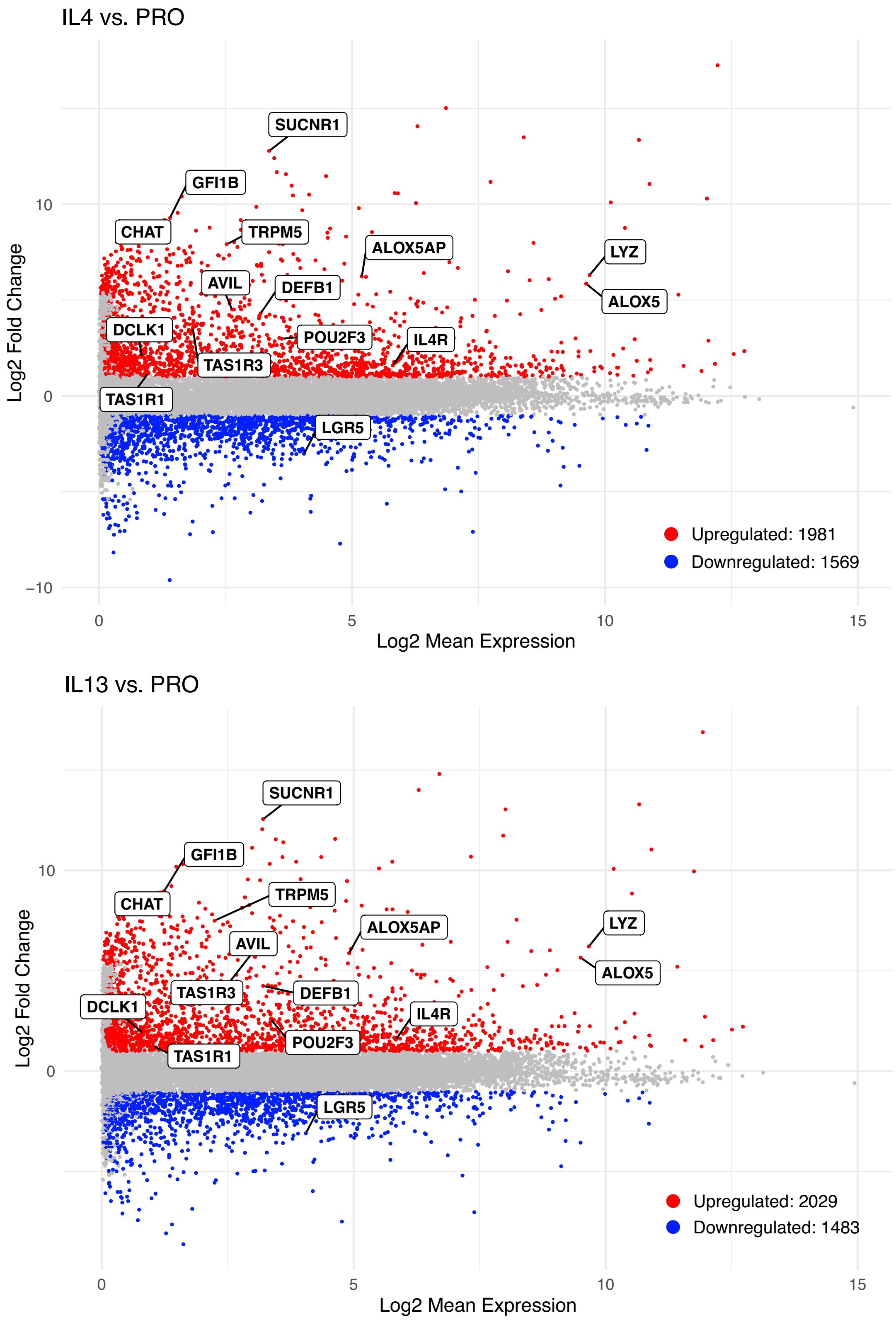

Supplementary Figure 3. MA plot illustrating gene expression changes in response to IL-4 or IL-13. MA plot depicting gene expression changes in response to IL-4 and IL-13 supplemented medium compared to PRO medium. Genes with no significant changes are centered around y = 0, while upregulated genes are plotted above the line (red) and downregulated genes below (blue). Gray dots represent genes with no significant change. ($|FC| \geq 2$, FDR p-value < 0.05).
