# Supplementary material for: Generation and functional characterization of tuft cells in non-human primate pancreatic ducts through organoid culture systems
Source: Front Cell Dev Biol. 2025 May 6;13:1593226. doi: 10.3389/fcell.2025.1593226 (PMC12089129; doi:10.3389/fcell.2025.1593226)
Supplement: Supplementary file 5 [file DataSheet1.pdf]

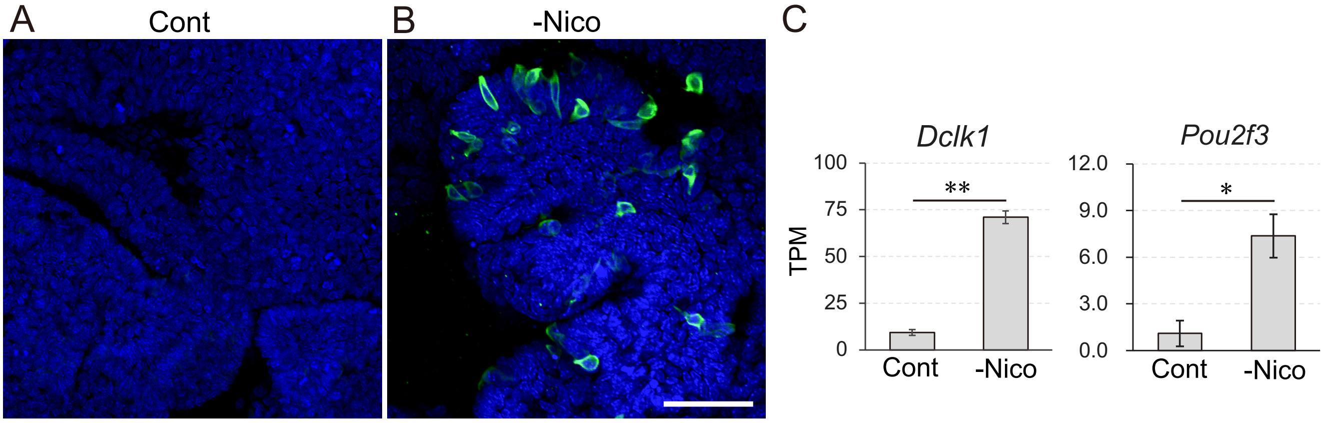

Supplementary Figure 1. Tuft cell differentiation is induced by withdrawal of nicotinamide in mouse pancreatic ductal organoids. Immunofluorescence staining of Dclk1 (green) in mouse pancreatic ductal organoids before **(A)** and after removing nicotinamide for 5 days **(B)**. Nuclei were stained with Hoechst 33342 (blue). Scale bars: 50 µm. Gene expression of tuft cell markers, *Dclk1* and *Pou2f3*, in mouse pancreatic ductal organoids before and after removing nicotinamide for 10 days **(C)**. Data are presented as means ± SEM ($n = 3$). *$P < 0.05$, **$P < 0.01$. Statistical significance was assessed using Welch's *t*-test.
