# Supplementary material for: Generation and functional characterization of tuft cells in non-human primate pancreatic ducts through organoid culture systems
Source: Front Cell Dev Biol. 2025 May 6;13:1593226. doi: 10.3389/fcell.2025.1593226 (PMC12089129; doi:10.3389/fcell.2025.1593226)
Supplement: Supplementary file 6 [file DataSheet5.pdf]

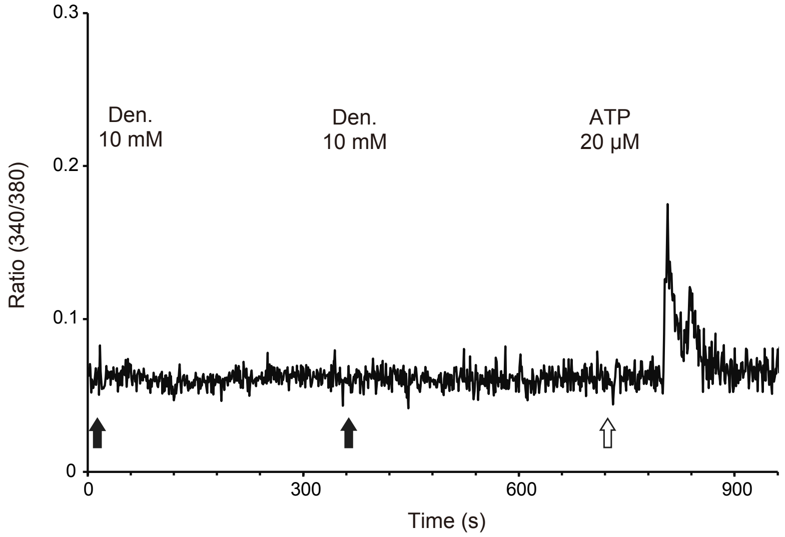

Supplementary Figure 5. Calcium response of pancreatic ductal organoids cultured in PRO medium to a bitter compound. A representative waveform of a cell derived from PRO medium-cultured organoids exposed to 10 mM denatonium benzoate (black arrows) is shown. ATP (20 μM, white arrow) was used to confirm cell viability.
